# A case of hypomania during nicotine cessation treatment with bupropion

**DOI:** 10.1186/1940-0640-8-22

**Published:** 2013-12-21

**Authors:** Karine Giasson-Gariépy, Didier Jutras-Aswad

**Affiliations:** 1 Research Center, Centre Hospitalier de l’Université de Montréal, Montreal, Quebec, Canada; 2 Department of Psychiatry, Université de Montréal, Montreal, Quebec, Canada

**Keywords:** Nicotine, Smoking cessation, Bupropion, Antidepressant, Hypomania, Venlafaxine

## Abstract

Antidepressants can increase the spontaneous risk of hypomania or mania when used for treatment in affective disorders. When prescribed as an antidepressant, bupropion is generally considered to have a lower relative risk of inducing mood shifts. We describe the case of a 67-year-old man known for dysthymic disorder in remission on quetiapine and venlafaxine who experienced a first lifetime episode of hypomania with the introduction of bupropion SR for smoking cessation. To the best of our knowledge, this is the first case report of bupropion-induced mood shift when used specifically for nicotine cessation in a nonbipolar patient. This case highlights the need for clinicians who prescribe bupropion for smoking cessation to perform regular and systematic mood follow-ups during treatment. These follow-ups could even be more important when bupropion is selected to quit smoking in a patient already taking an antidepressant.

## Background

Bupropion SR is a dopamine/norepinephrine reuptake inhibitor licensed for use as a smoking cessation aid. Compared with other antidepressants, bupropion is generally considered to have a lower relative risk of inducing mood shifts [1], however there are no controlled studies specifically addressing this risk when prescribed for nicotine dependence treatment. Secondary mania induction in unipolar depression treatment with bupropion has been infrequently reported in the literature, but little is known about the potential for mood shifts when bupropion is prescribed as a smoking cessation aid. We describe here a patient who experienced a first lifetime episode of hypomania with the introduction of bupropion SR for smoking cessation. To the best of our knowledge, this is the first case report of bupropion-induced mood shift when used specifically for smoking cessation in a nonbipolar patient.

## Case presentation

Mr. X was a 67-year-old man with nicotine dependence (45 packs-years), alcohol dependence in early full remission, and dysthymic disorder in remission. He was maintained on venlafaxine XR (225 mg per day) and quetiapine XR (50 mg per day + an additional 25 mg twice daily as needed) with no recent treatment regimen modification. Past psychiatric history was significant for recurrent unipolar major depressive episodes and cocaine abuse. Past substance use history included daily alcohol use since age 35. The number of standard drinks per day increased over the years but averaged 14–20 in the two years preceding his sobriety period. Cocaine was used once per month from the age of 35 to 45 and approximately two times per year thereafter until his sobriety period. He reported no other recent regular substance use. The patient had been stable psychiatrically with no alcohol or cocaine use for seven months. There was no personal or family history of bipolar disorder, but prior long-term substance use history remains a potential confounding factor.

For smoking cessation, bupropion SR coupled with nicotine replacement therapy (NRT) (14 mg patch daily) and therapeutic groups were used. In March 2012, bupropion SR (150 mg per day) was prescribed for three days and a preplanned quit date was set for day 4. As the treatment began, the patient noticed some feelings of excitement, which were amplified when the dose was increased to 150 mg twice daily on the fourth day of treatment. From that point on, he reported the onset of euphoria, racing thoughts, and decreased need for sleep. He subsequently relapsed to alcohol and cocaine use (on days 7 and 8) with minimization of the consequences of substance use. He mentioned no suicidality or psychotic symptoms. The patient met his therapist during the smoking cessation group on day 5, who noticed a change in the patient’s affect and describing him as more anxious, tense, and keyed up. Subjectively, on that day, the patient noticed being more anxious and having less ability to concentrate, which could be early nicotine withdrawal symptoms or early hypomanic symptoms. When seen by his psychiatrist 11 days after treatment initiation, the patient had decided to stop substance use and bupropion for two days. He was getting back to his baseline level, reporting some residual anxiety. Venlafaxine and NRT were maintained, while his quetiapine XR dose was increased to 100 mg per day regularly; bupropion was discontinued. One week later, the patient’s mood was stable, and he had not used any substances.

## Discussion

The timing of symptoms suggests an association between bupropion initiation and mood shift in this patient. Bupropion can induce mood switches in bipolar depression, but possibly less frequently than other antidepressants [2]. There are only a few cases reported of bupropion-induced mood shifts in unipolar disorders and of secondary mania induction [3-5]. To our knowledge, bupropion-induced polarity changes in nonbipolar patients have not been reported during the management of smoking cessation, except for a case of mania after abrupt treatment discontinuation [6].

A clinical interaction between bupropion and venlafaxine could also explain the hypomania. Venlafaxine is a serotonin/norepinephrine reuptake inhibitor. When used as an adjunct in bipolar depression, it is associated with an increased risk of mood shifts compared with bupropion [1]. An open-label study found a 2.5-fold increase in plasma levels of venlafaxine when bupropion is added, possibly via CYP2D6 inhibition [7]. A case series mentioned the need to decrease venlafaxine dose when bupropion was added for the treatment of major depressive disorder in order to diminish serotonergic side-effects [8]. Despite this known pharmacokinetic interaction, the evidence of its clinical implication remains limited. Combining therapeutic doses of these antidepressants with overlapping mechanisms of action and pharmacokinetic interactions could also enhance noradrenergic stimulating effects, which could contribute to the emergence of hypomanic symptoms.

Other contributing factors first include nicotine withdrawal symptoms, which include irritability, restlessness, insomnia, anxiety, and poor concentration. Nicotine withdrawal symptoms could have potentiated or predisposed the patient to this mood shift. We do not consider the patient’s substance use to be a contributing factor to his mood shift, since the hypomanic symptoms noticeably took place before the cocaine/alcohol use. However, his past substance use disorder could have been a predisposing factor, as it has been associated with an increased risk of antidepressant-induced mania/hypomania in bipolar patients [9].

## Conclusions

Although there are several hypotheses for the hypomanic episode, our findings suggest the need to carefully monitor for mood shifts when bupropion is prescribed as a smoking cessation aid, even in nonbipolar patients and in particular when combined with other antidepressants. Smoking cessation represents a sensitive period during which many factors can alter mood (e.g. nicotine withdrawal, NRT, the stress of quitting smoking). These factors may modify the risk of mood shifts associated with bupropion.

Although the benefits of medication-assisted smoking cessation are clear, there is a need for frequent and systematic monitoring of mood symptoms during treatment with bupropion in the context of smoking cessation, even in patients not previously diagnosed with bipolar disorder. For patients who are already receiving antidepressant treatment, selecting bupropion over other smoking-cessation strategies should take into account the risk of mood shifts and interactions.

## Consent

Written informed consent was obtained from the patient for publication of this case report.

## Competing interests

This work was supported by the CHUM Department of Psychiatry; Université de Montréal Department of Psychiatry; and the CHUM Research Center (DJA). The authors have no conflicts of interest with this case study. Dr. Jutras-Aswad has received research/education grant support from Pfizer, Janssen, Bristol-Myers Squibb, Mylan and Reckitt Benckiser Pharmaceuticals, as well as presentation honoraria from Janssen, consultant honoraria from Merck and grant support from the CHUM Department of Psychiatry, Université de Montréal Department of Psychiatry and the CHUM Research Center. The authors are solely responsible for the writing of this case study.

## Authors’ contributions

DJA conceived the case report. DJA and KGG drafted the manuscript. Both authors read and approved the final manuscript.
